# Polish Translation and Validation of the Tinnitus Handicap Inventory and the Tinnitus Functional Index

**DOI:** 10.3389/fpsyg.2016.01871

**Published:** 2016-11-29

**Authors:** Małgorzata Wrzosek, Eugeniusz Szymiec, Wiesława Klemens, Piotr Kotyło, Winfried Schlee, Małgorzata Modrzyńska, Agnieszka Lang-Małecka, Anna Preis, Jan Bulla

**Affiliations:** ^1^Department of Logic and Cognitive Science, Adam Mickiewicz UniversityPoznań, Poland; ^2^ENT Department, University of Medical SciencesPoznań, Poland; ^3^ENT Private PracticeGdynia, Poland; ^4^Audiology and Phoniatrics Clinic, Nofer Institute of Occupational Medicine in ŁódźŁódź, Poland; ^5^Department of Psychiatry and Psychotherapy, University of RegensburgRegensburg, Germany; ^6^Institute of Acoustics, Adam Mickiewicz UniversityPoznań, Poland; ^7^Department of Mathematics, University of BergenBergen, Norway

**Keywords:** Tinnitus Handicap Inventory, THI, Tinnitus Functional Index, TFI, Polish, adaptation

## Abstract

**Objective:** The need for validated measures enabling clinicians to classify tinnitus patients according to the severity of tinnitus and screen the progress of therapies in our country led us to translate into Polish and to validate two tinnitus questionnaires, namely the Tinnitus Handicap Inventory (THI) and the Tinnitus Functional Index (TFI).

**Design:** The original English versions of the questionnaires were translated into Polish and translated back to English by three independent translators. These versions were then finalized by the authors into a Polish THI (THI-Pl) and a Polish TFI (TFI-Pl). Participants from three laryngological centers in Poland anonymously answered the THI-Pl (*N* = 98) and the TFI-Pl (*N* = 108) in addition to the Polish versions of the Center for Epidemiologic Studies Depression Scale as a measure of self-perceived level of depression, and the Satisfaction With Life Scale to assess self-perceived quality of life. Both were used to determine discriminant validity. Two Visual Analog Scales were used to measure tinnitus annoyance and tinnitus loudness in order to determine convergent validity.

**Results:** Similar to the original version of the THI, the THI-Pl showed a high internal consistency (Cronbach’s α = 0.93). The exploratory factor analysis revealed that the questionnaire has a three-factorial structure that does not correspond to the original division for functional, catastrophic, and emotional subscales. Convergent and discriminant validities were confirmed. The TFI-Pl showed high internal consistency (Cronbach’s α = 0.96) with the reliability ranging from 0.82 to 0.95 for its different subscales. Factor analysis confirmed an eight-factorial structure with factors assigning all items to appropriate subscales reported in the original version of the questionnaire. Discriminant and convergent validities were also confirmed for the TFI-Pl.

**Conclusion:** We translated and validated the Polish versions of the THI and the TFI to make them suitable for clinical use in Poland.

## Introduction

Tinnitus (“ringing in the ears”) is described as the perception of sound without any external stimulation. Chronic tinnitus is a common condition, affecting around 10% of the general population, and for some people this condition is debilitating (Langguth et al., 2011). As for the Polish adult population, Skarżyński et al. (2000) estimated that 20% experienced tinnitus lasting more than 5 min, around 5% were affected by chronic tinnitus, and for 4% (almost 1.6 million adults) tinnitus caused severe annoyance. Moreover, Polish children are affected by this problem as well. The study by Raj-Koziak et al. (2011) reports that more than 5% of 55201 7-year-old children tested reported their perception of tinnitus to be often or very often.

Tinnitus can seriously affect quality of life and, in extreme cases, even lead to suicide (Jastreboff and Hazell, 2004). Among tinnitus related comorbidities we can distinguish anxiety (Udupi et al., 2013; Kehrle et al., 2016), depression (Langguth et al., 2011), or sleep disorders (Crönlein et al., 2016). Tinnitus may be closely linked to hearing loss (Martines et al., 2010; Schecklmann et al., 2012). Different therapeutic approaches offer the use of hearing aids, tinnitus maskers, or tinnitus instruments that combine both (Vernon and Meikle, 2003); counseling sessions (e.g., cognitive behavior therapy, see Cima et al., 2014); counseling combined with the use of sound generators [e.g., Tinnitus Retraining Therapy (TRT), see Jastreboff and Hazel, 1993]; relaxation techniques (e.g., Mindfulness Based Stress Reduction, see Roland et al., 2015); neuromodulation (e.g., the Acoustic Coordinated Reset Neuromodulation, see Tass et al., 2012); or brain stimulation (e.g., Repetitive Transcranial Magnetic Stimulation Treatment, see Folmer et al., 2015). There are many possible options for treatment; however, none of these provides immediate and constant relief for tinnitus (for further details see, e.g., Baguley et al., 2013; Maldonado Fernández et al., 2015).

At the moment, no valid and standardized questionnaire for the assessment of tinnitus is available in Poland. As of yet, clinicians in Poland rely on self-made, non-validated translations of the original version of the questionnaire, which limits the reliability of patient assessment, but also prevents the comparison of therapeutic outcomes with other clinics using validated instruments.

We decided to translate and validate the Tinnitus Handicap Inventory (THI) and the Tinnitus Functional Index (TFI) developed by Newman et al. (1996) and Meikle et al. (2012), respectively. Our choice of the THI was based on two observations: firstly, it is a well-known questionnaire that has been extensively used in clinical and scientific practice to measure tinnitus distress and to test potential reduction of the THI score as a consequence of applied therapy (see, e.g., Landgrebe et al., 2010; Shekhawat et al., 2013; Roland et al., 2015; Wilson et al., 2015; Wise et al., 2015; Zobay et al., 2015). It was determined that a clinically meaningful change is achieved with a reduction of 20 or more points of the total score of the THI (Newman et al., 1996, 1998; Fackrell et al., 2014). Secondly, the THI is integrated into the Tinnitus Research Initiative database, which contains standardized data collected from different tinnitus research centers and countries (Landgrebe et al., 2010). A validated Polish version of the tool would enable clinicians not only to classify tinnitus patients according to severity, but would also create a possibility for Polish scientists to contribute to international research projects.

Our second choice—the TFI—was based on its unique feature; precisely, the possibility to evaluate therapeutic outcomes (Meikle et al., 2012). In addition, this questionnaire may be used by clinicians and researchers to classify patients according to tinnitus severity. Nevertheless, our goal was to provide to Polish ENT centers a tool which enables the evaluation of the effectiveness of applied treatments (see, e.g., Krings et al., 2014; Folmer et al., 2015; Overdevest et al., 2015; Roland et al., 2015; Wilson et al., 2015; Fackrell et al., 2016).

## Materials and Methods

### Participants

The study was performed in three clinics in Polish cities—namely Poznań, Gdańsk and Łódź —where paper versions of the questionnaires were filled out anonymously by 98 patients (for the THI-Pl) and 108 patients (for the TFI-PI) who reported tinnitus as either a primary complaint or secondary complaint after hearing loss (defined by hearing loss exceeding 25 dB HL for at least one of the frequencies tested in the audiological pre-interview). Ten of 108 patients tested with the TFI-Pl questionnaire did not fill out the THI-Pl. Participation was voluntary, all participants gave oral consent before filling out the questionnaire, and data was stored and analyzed completely anonymously. The sample was heterogeneous and included a large variety of patients visiting our clinics. This range included, for example, patients with different degrees of hearing loss and self-reported tinnitus severity visiting the ENT doctor for the first time, but also those who had already undergone particular treatments and patients currently in the therapeutic process (Poznań: mainly TRT or electrostimulation; Gdańsk: TRT; Łódź: TRT, counseling sessions). A study information sheet was provided and all volunteers were informed about the aim of the study as well as the estimated time for completing the questionnaires. The participants filled out the questionnaires while waiting for their consultation with an ENT specialist. Descriptive measures of patients who filled in the THI-Pl and TFI-Pl questionnaires are shown in Supplementary Tables S1 and S2, respectively.

### Tinnitus Handicap Inventory

The THI (Newman et al., 1996) is a self-reported measure consisting of 25 items divided into three subscales: functional (11 items measuring the functional aspects of tinnitus such as mental, social/occupational, and physical functioning), catastrophic (five items reflecting catastrophic responses to tinnitus, including depression, and sleep disturbance), and emotional (nine items representing affective responses to tinnitus). There are three possible answers to each item (and 25 items in total): “yes” (four points), “sometimes” (two points), and “no” (zero points). Scores are calculated for the THI total scale (range 0–100 points) as well as for the three subscales: functional (THIf), catastrophic (THIc), and emotional (THIe)—ranges 0–44, 0–20, and 0–36 points, respectively). It is noteworthy that some researchers proposed a unifactorial structure of the questionnaire, with no division for subscales (Baguley and Andersson, 2003; Fackrell et al., 2014).

The THI has been widely validated and translated, for example, into Danish (Zachariae et al., 2000), Korean (Kim et al., 2002), Italian (Monzani et al., 2008), Chinese (Kam et al., 2009), German (Kleinstäuber et al., 2015), Persian (Jalali et al., 2015), and Russian (Oron et al., 2015).

### Tinnitus Functional Index

The TFI (Meikle et al., 2012) is a self-report measure used for the evaluation of tinnitus severity, measuring clinically important changes. It consists of 25 items divided into 8 subscales addressing different domains of tinnitus severity: intrusive (three items, TFIint), sense of control (three items, TFIsoc), cognitive (three items, TFIcog), sleep (three items, TFIsleep), auditory (three items, TFIaud), relaxation (three items, TFIrelax), quality of life (four items, TFIqol) and emotional (three items, TFIem). The TFI-Pl uses a 10-point scale in the range 0–10 or 0–100%. The scores are calculated for the total scale and all subscales (range 0–100 each). Detailed scoring instructions are provided by the authors (Meikle et al., 2012).

The TFI is currently translated and validated by scientists from Holland (Rabau et al., 2014) and England (Fackrell et al., 2016).

### Translation Procedure

The original versions of the questionnaires were translated into Polish by the authors fluent in English. These authors agreed to the translated version being further forwarded to three independent translators who performed translations back into English. Two of these translators were native English speakers and one was an English teacher who studied English philology. They were unfamiliar with the original version of the questionnaire. After comparing the original and the translated versions, the authors constructed the final versions of the THI-Pl and TFI-Pl (see “Supplementary Material 1” which displays the Polish versions of the THI and information on how to obtain the TFI), based on a simple frequency criterion. More precisely, when discrepancies between the three translations provided were observed, the authors chose the version preferred by two of the three translators. Other forms of discrepancies were not observed. The questionnaires were pre-tested by the authors themselves and with hospital staff who volunteered for this task.

### Additional Measures

Participants were additionally asked to complete the Polish version of The Centre for Epidemiological Studies Depression Scale (CES-D; Radloff, 1977; Polish adaptation: Dojka et al., 2003; Kaniasty, 2003; Ziarko et al., 2013) and the Polish version of The Satisfaction With Life Scale (SWLS; Diener et al., 1985; Pavot and Diener, 1993; Polish adaptation: Juczyński, 2001). We also used two versions of the Visual Analog Scale as a measure of self-perceived tinnitus annoyance and tinnitus loudness.

The CES-D is a tool which is free of charge and widely used in clinical practice to estimate the number and intensity of depressive symptoms after the diagnosis of depression (Ziarko et al., 2013). It consists of 25 items divided into four subscales measuring: (1) depressive affect (depressive mood), (2) lack of positive affect (well-being), (3) somatic symptoms and inhibition of activity (somatic symptoms), and (4) attitude to other people (intrapersonal affect) (Radloff, 1977; Ziarko et al., 2013). The structure of the Polish version of the tool is internally compliant with the theoretical assumptions and its temporal stability was confirmed in the longitudinal studies (Ziarko et al., 2013).

The SWLS assesses self-perceived quality of life. The tool includes five statements with which patients have to express their degree of agreement (1–“I totally disagree,” 7–“I totally agree”). The total score of the Polish and original version of this tool is a general indicator of a participant’s satisfaction with their life (Juczyński, 2001).

The Visual Analog Scales (VAS; assessing self-perceived tinnitus anxiety and tinnitus loudness) were chosen, because they are considered to be valid and effective tools for measuring reductions in tinnitus severity in people with chronic tinnitus (Adamchic et al., 2012). The Visual Analog Scale was used in order to ascertain the severity of tinnitus (tinnitus annoyance and tinnitus loudness). The VAS scales consisted of two 10 cm lines with marked endpoints. There were two faces drawn: a smiling one indicating lack of annoyance or no perception of tinnitus (painted under the left endpoint of a line) and a sad one indicating extreme annoyance or extremely loud tinnitus (painted under the right endpoint of a line).

### Statistical Analysis

Statistical analyses of data were performed with the Statistical Package for Social Sciences (v22; SPSS, Inc., Chicago, IL, USA). Since the majority of our samples were non-Gaussian (indicated by the Shapiro–Wilk test), we relied mainly on non-parametric techniques. More specifically, whenever the normality assumption was rejected, we used the Wilcoxon–Mann–Whitney for comparisons of two (unpaired) groups. Alternatively, the *t*-test was preferred for Gaussian samples. Similarly, the Kruskal–Wallis test served to compare larger numbers of groups when the normality hypothesis was rejected. For the Gaussian sample, we used a one-way ANOVA. Spearman’s rank correlation coefficient (rho) was used for measuring associations. Internal consistency was evaluated by measuring Cronbach’s alpha coefficient in order to assess questionnaire reliability and the item-total correlation. The Kaiser-Meyer-Olkin (KMO) Measure of Sampling Adequacy and Bartlett’s Test of Sphericity were assessed to assure that our data was eligible for our exploratory factor analysis. Correlations between the factors were calculated, and factor analysis was carried out using the oblique Oblimin rotation. In addition, two orthogonal rotations (Varimax, Quartimax) were investigated. For increased robustness, we selected the Unweighted Least Squares method as an estimation technique. Confirmatory factor analysis (CFA) was performed using IBM SPSS AMOS 22. Statistical significance was set for *p*-values smaller than 0.05.

In order to determine the discriminant validity, we asked participants to complete the Polish version of The Centre for Epidemiological Studies Depression Scale (CES-D; Radloff, 1977; Polish adaptation: Dojka et al., 2003; Kaniasty, 2003; Ziarko et al., 2013) and the Polish version of The Satisfaction With Life Scale (SWLS; Diener et al., 1985; Pavot and Diener, 1993; Polish adaptation: Juczyński, 2001). We assumed that discriminant validity would be confirmed when at most moderate (ρ < 0.6) correlations between the THI and TFI total score and CES-D and SWLS scores were observed (Newman et al., 1996; Fackrell et al., 2014).

To access the convergent validity, we used two versions of the Visual Analog Scale as a measure of the self-perceived tinnitus annoyance and tinnitus loudness. We assumed that convergent validity would be confirmed when at least strong correlations (ρ > 0.6) between the THI and TFI total score and VAS annoyance and VAS loudness scores were observed.

Five TFI-Pl questionnaires from two female and three male respondents with seven or more omitted responses were removed from the analysis, according to the recommendation by Meikle et al. (2012). We did not have to exclude any THI-Pl questionnaires. Thus, the final number of questionnaires retained was *N* = 98 and *N* = 103 for the THI and TFI analysis, respectively. Note that our THI-Pl sample contained a total of 35 missing observations, and the TFI-Pl sample contained a total of 22 missing observations (after the exclusion of five participants), resulting from questions skipped by responders. Since this constitutes only ∼1% of the data, we applied a mean-replacement for reliability analysis as well as a factor analysis.

## Results

### The Polish version of the THI

#### Descriptive Statistics, Intergroup Differences, and Correlations

The average age of patients who filled in the THI-Pl questionnaire was 51.72 years (*SD* = 13.06) and the mean duration of tinnitus was 6.56 years (*SD* = 11.64). Responses given to particular items of the THI-Pl are presented in Supplementary Table S3. **Table 1** shows average scores obtained by participants for the THI-Pl, VAS scales, CES-D, and SWLS. **Table 2** summarizes correlations between the different scales. The highest correlations were found for the THI total score and VAS annoyance. The relation between the THI total score (or its subscales) and gender was not significant, as shown by the Wilcoxon–Mann–Whitney test. In addition, the Kruskal–Wallis test did not reveal any significant differences for THI total score (or its subscales) resulting from variations in tinnitus pitch, localization, or character. The Wilcoxon–Mann–Whitney test showed significant influence of hearing loss on the total THI score (*p* = 0.044). Participants subject to hearing loss obtained higher scores than normal hearing patients (41.6 vs. 33.4). There were no significant correlations between the THI-Pl and age or duration of tinnitus.

**Table 1:** Average scores and their standard deviations for THI-Pl and additional measures obtained from the Polish participants.

	Scale							
	**THI-Pl**	**THIf**	**THIc**	**THIe**	**VASa**	**VASl**	**CES-D**	**SWLS**

Mean	38.6	17.7	8.8	12.1	48.9	50.0	15.6	21.5
*SD*	22.9	10.3	5.1	9.5	26.3	26.2	10.6	5.8

*THI, Tinnitus Handicap Inventory; Pl, Polish; f, functional; c, catastrophic; e, emotional; VAS, Visual Analog Scale; a, annoyance; l, loudness; SWLS, The Satisfaction With Life Scale; CES-D, The Centre for Epidemiological Studies Depression Scale.*

**Table 2:** Correlations (Spearman’s rho) between the THI-Pl and other measures used in the adaptation procedure.

Measure	VASa	VASl	CES-D	SWLS
THI-Pl	0.799^∗∗∗^	0.610^∗∗∗^	0.528^∗∗∗^	-0.300^∗∗^

*^∗∗∗^*p* < 0.001; ^∗∗^*p* < 0.01; THI, Tinnitus Handicap Inventory; Pl, Polish; VAS, Visual Analog Scale; a, annoyance; l, loudness; CES-D, The Centre for Epidemiological Studies Depression Scale; SWLS, The Satisfaction With Life Scale.*

Convergent validity was confirmed by strong positive Spearman’s correlations for the total THI score and the VAS annoyance and loudness scales. Discriminant validity was assessed by weak negative correlations (ρ < 0.4) for the SWLS and moderate positive correlations (ρ < 0.6) for the CES-D total score.

#### Internal Consistency

The THI-Pl has the same high internal consistency reliability as the original version of the questionnaire (α = 0.93; from Newman et al., 1996, which serves as a reference for all statistical measures in the following as well, if not stated otherwise). **Table 3** presents the corrected item total correlation. Further details concerning Cronbach’s alpha coefficient if an item is deleted are included in Supplementary Table S4. Items 2, 19, and 24 had the lowest possible corrected item total correlation, with values lower than 0.4–0.30, 0.25, and 0.27, respectively. The analysis revealed that the removal of these items would only slightly elevate Cronbach’s alpha coefficient from α = 0.933 to α = 0.935. We therefore decided to preserve the original number of items in the questionnaire. For the other 22 items, the corrected item total correlation was high (*r* = 0.6). When compared to the original version of questionnaire, internal consistency reliability was slightly lower for the functional subscale (α = 0.83 vs. α = 0.86) and slightly higher for the catastrophic (α = 0.70 vs. α = 0.68) and emotional (α = 0.90 vs. α = 0.87) subscales. Supplementary Table S5 lists the reliability coefficients of the THI-Pl and other adaptations. The last two columns report the average internal consistency reliability of all adaptations excluding the Polish and the original English version and the sample standard deviation. Cronbach’s alpha determined for the Polish adaptation fell within the estimated 95% quantile of all questionnaires as presented in Supplementary Table S5.

**Table 3:** Corrected item total correlation for all items of the THI-Pl.

Scale/Item	Corrected item-total correlation
F 1	0.706
F 2	0.303
E 3	0.514
F 4	0.514
C 5	0.673
E 6	0.668
F 7	0.405
C 8	0.481
F 9	0.720
E 10	0.682
C 11	0.486
F 12	0.720
F 13	0.627
E 14	0.711
F 15	0.536
E 16	0.694
E 17	0.628
F 18	0.495
C 19	0.246
F 20	0.674
E 21	0.713
E 22	0.743
C 23	0.632
F 24	0.266
E 25	0.748

*F, functional; E, emotional; C, catastrophic.*

#### Factor Analysis

We started with an exploratory factor analysis, assuming the original structure of the questionnaire (Newman et al., 1996) with three factors. All factors were moderately correlated with each other, as can be seen in the lower part of **Table 4**, supporting the choice of an oblique rotation. The first main factor with an eigenvalue of 10.2 explained 41.0% of the variance; the second with an eigenvalue of 1.9–7.5% and the third with an eigenvalue of 1.4–5.5% together explained 54.1% of the total variance. Supplementary Figure S1 shows a scree plot of factors with corresponding eigenvalues. On the one hand, the first factor is dominant, which at first glance supports a unifactorial questionnaire structure. **Table 4** represents the rotated factor loadings of the described solution and the factor correlations. Investigation of the factor loadings and their corresponding eigenvalues led us to three conclusions. Firstly, three factors contribute to the loadings of the 25 items, but these factors do not correspond to the subscales of the questionnaire. Except for three items, all of these loadings can be considered important as they have values greater than 0.4 (Floyd and Widaman, 1995; Baguley and Andersson, 2003). In addition, the remaining items carry loadings higher than 0.27 and two of these are greater than 0.3. Secondly, of the first factor loads on 15 items, six belong to the emotional subscale, four to the catastrophic subscale, and five to the functional one. In our opinion, these items may be considered to be referring to the impact of tinnitus on everyday functioning and the emotional state of the patient. Five questions are loaded by the second factor, four of which originally belonged to the functional and one to the emotional subscale. These items could be viewed as describing the aspect of “helplessness” resulting from the perception of tinnitus. The third factor also loads five items, two belonging to the functional, two to the emotional, and one to the catastrophic subscale. Four of these questions refer either to relationships with other people or to social activities. Item number 12 concerns satisfaction with life (“Does your tinnitus make it difficult for you to enjoy life?”) and, in our belief, can be linked with satisfaction from social relations. Taking into consideration loadings greater than 0.3, only three items (5, 22, and 23) are double-loaded. Two of them—item 5 (“Because of your tinnitus, do you feel desperate?”) and item 23 (“Do you feel that you can no longer cope with your tinnitus?”)—are additionally loaded by the third factor, which seems to be appropriate in the context of “helplessness.” The question “Does your tinnitus make you feel anxious?” (item 22) is loaded by the third factor, but also by the first one. Since this item refers to the emotional state of the patient, an observation of double-loading seems to be justified.

**Table 4:** Rotated factor loading matrices of the predefined-factor models.

	Factor		
Scale/Item	1	2	3
	10.25	1.88	1.38
E 16	**0.672**		
F 15	**0.641**		
F 20	**0.637**		
F 7	**0.627**		
C 8	**0.534**		
F 18	**0.516**		
C 23	**0.511**		0.359
E 25	**0.494**		
F 1	**0.494**		
E 10	**0.477**		
E 14	**0.465**		
E 6	**0.447**		
C 19	**0.435**		
C 5	**0.425**		0.355
E 3	**0.280**		
E 17		**-0.886**	
F 13		**-0.744**	
F 9		**-0.586**	
F 2		**-0.472**	
F 12		**-0.452**	
E 21			**0.699**
F 24			**0.556**
E 22	0.464		**0.473**
F 4			**0.319**
C 11			**0.310**

**Correlations**			

Factor 1	1.000	**-**0.433	0.317
Factor 2	**-**0.433	1.000	**-**0.442
Factor 3	0.317	**-**0.442	1.000

*Eigenvalues are presented below the names of factors. Lower part of the table shows correlations between the factors. Loadings > 0.30 displayed (with exception of Item 3). Loadings assigned to particular factors in bold; F, functional; E, emotional; C, catastrophic.*

Thirdly, the first factor alone explains less than half of the total variance. Taking this and the factor loadings of the second and third factors into account, it may be advisable to consider at least a two- or even three-factorial structure for attaining approximately 50% explained variation.

Moreover, we also investigated the effect of using orthogonal rotations for exploratory purposes (Supplementary Table S6 reports the results). Then the results changed substantially: on the one hand, the Varimax solution suggests a structure similar to that obtained by the Oblimin solution. On the other hand, the Quartimax rotation leads to one main factor carrying the highest load for 22 of the 25 items, suggesting a unifactorial structure.

Lastly, we carried out a CFA for investigating if we can confirm the original three-factorial structure suggested by Newman et al. (1996). Some of the values of our fit indices were not too far from those obtained by Kleinstäuber et al. (2015); for example, Kleinstäuber et al. (2015) obtained an RMSEA of 0.060, whereas our corresponding value equals 0.084. However, the overall results did not permit us to conclude an acceptable fit with the data, which is also in line with Kleinstäuber et al. (2015). Supplementary Table S14 presents details of the estimation results. Due to our comparably low sample size and the resulting lower reliability of all χ^2^-based statistics, we refrained from investigating further modifications of our factor model.

### The Polish version of the TFI

#### Descriptive Statistics, Intergroup Differences, and Correlations

Supplementary Table S7 presents responses to particular items of the TFI-Pl. **Table 5** shows the average scores obtained by participants for the TFI-Pl, VAS scales, CES-D, and SWLS. **Table 6** summarizes the correlations between the different scales (correlations between THI-Pl and TFI-Pl are included). Lastly, Supplementary Table S8 presents more details on correlations of all THI and TFI scales with other measures.

**Table 5:** Average scores and their standard deviations for TFI-Pl, the original version of the TFI (values in brackets), and additional measures obtained from the Polish participants.

Scale	Mean	*SD*
TFI	46.7 (54.4)	22.5 (24.7)
TFIint	53.1 (67.8)	25.9 (24.3)
TFIsoc	64.0 (64.7)	27.0 (25.0)
TFIcog	40.2 (48.0)	25.0 (29.0)
TFIsleep	42.6 (51.5)	31.5 (35.3)
TFIaud	40.0 (53.1)	29.3 (30.2)
TFIrelax	50.5 (59.9)	28.9 (30.3)
TFIqol	36.4 (46.3)	29.4 (29.9)
TFIem	45.6 (47.2)	29.0 (31.8)
VASa	4.8	2.6
VASl	4.9	2.6
CES-D	15.2	10.3
SWLS	21.8	5.8

*TFI, Tinnitus Functional Index; int, intrusive; soc, sence of control; cog, cognitive; aud, auditory; relax, relaxation; qol, quality of life; em, emotional; VAS, Visual Analog Scale; a, annoyance; l, loudness; CES-D, The Centre for Epidemiological Studies Depression Scale; SWLS, The Satisfaction With Life Scale.*

**Table 6:** Correlations (Spearman’s rho) for TFI-Pl and other measures used in the adaptation procedure.

Measure	THI-Pl	VASa	VASl	CES-D	SWLS
TFI-Pl	782^∗∗∗^	0.837^∗∗∗^	0.761^∗∗∗^	0.361^∗∗∗^	**-**0.223^∗^

*^∗∗∗^*p* < 0.001; ^∗^*p* < 0.05; TFI, Tinnitus Functional Index; THI, Tinnitus Handicap Inventory; Pl, Polish; VAS, Visual Analog Scale; a, annoyance; l, loudness; CES-D, The Centre for Epidemiological Studies Depression Scale; SWLS, The Satisfaction With Life Scale.*

We did not discover any impact of gender or hearing loss on the TFI-Pl score or its subscales (all *p*-values from *t*-test were non-significant). The Wilcoxon–Mann–Whitney test showed significant influence of hearing loss presence on the scores obtained in the auditory, quality of life, and emotional subscales (*p* = 0.017, *p* = 0.020, and *p* = 0.024, respectively). The group with participants subject to hearing loss obtained higher scores than normal hearing patients. In addition, the Kruskal–Wallis test revealed significant differences for the quality of life (*p* = 0.03) and emotional (*p* = 0.049) subscales and tinnitus pitch as well as for the sense of control subscale and tinnitus characteristics (tonal vs. non-tonal tinnitus). The highest scores in both the quality of life and emotional subscales were obtained by participants whose tinnitus pitch corresponded to a sound of 125 Hz; the lowest score corresponded to 500 Hz. The lowest sense of control was reported for patients experiencing a mixed type of tinnitus. A one-way ANOVA did not confirm any significant differences for the TFI-Pl total score (or its subscales) resulting from variations in tinnitus localization or character. Significant differences were observed for the relaxation subscale and tinnitus pitch (*p* = 0.012). A tinnitus pitch of 125 Hz interfered with relaxation particularly strongly, whereas 250 Hz corresponded to the lowest score obtained on the relaxation subscale. Weak but significant correlations were found between the auditory subscale and age or duration of tinnitus.

Convergent validity was confirmed with strong positive Spearman’s correlations for the total TFI-Pl score and VAS loudness scale and very strong correlations with the VAS annoyance scale. Moreover, strong correlations were found between the THI-Pl and TFI-Pl. Discriminant validity was shown with no significant correlations for the SWLS and weak positive correlations for the CES-D. The lowest correlations were found for the TFI auditory subscale.

#### Internal Consistency

The TFI-Pl has almost the same high internal consistency reliability as the original version of the questionnaire (α = 0.96 vs. 0.97). Consistency reliability of the TFI subscales ranged from 0.83 (sense of control subscale) to 0.95 (emotional subscale). **Table 7** shows the corrected-item total correlation; Supplementary Table S9 section contains additional information about Cronbach’s alpha when a particular item is deleted. Supplementary Table S10 section presents Cronbach’s alpha coefficients for the TFI total and all subscales for the TFI-Pl and other adapted versions.

**Table 7:** Corrected item total correlation for all items of the TFI-Pl.

Item	Corrected item-total correlation
1	0.585
2	0.736
3	0.666
4	0.374
5	0.669
6	0.717
7	0.747
8	0.740
9	0.772
10	0.609
11	0.655
12	0.642
13	0.604
14	0.487
15	0.599
16	0.739
17	0.715
18	0.728
19	0.769
20	0.833
21	0.780
22	0.721
23	0.784
24	0.798
25	0.798

#### Factor Analysis

Similar to the THI, we carried out an exploratory factor analysis based on the oblique Oblimin rotation, since several factors showed moderate correlation (see lower part of **Table 8**). The resulting eigenvalues greater than or equal 1 indicated that five factors are sufficient for explaining the total variance (the fifth factor had an eigenvalue of 0.999). The first factor explained 52.7% of variance, the second 10.8%, the third 5.7%, and the two remaining 5.2% and 4.0%, respectively. The following three factors had eigenvalues of lower than 1 (0.87, 0.67, and 0.51), explaining altogether 8.2% of variance. Meikle et al. (2012) obtained similar results in the factor analysis of their first prototype of the TFI with 43 items, where the eigenvalues of factors five to eight were also lower than 1. However, these authors considered eight factors meaningful and decided to retain all of them. They then performed further confirmatory analysis of a second prototype as well as the final version of the TFI with a specified number (8) of factors. In their analysis of the final version, 79.5% of the total variance was explained, whereas in our case eight factors are explained 86.7%. It may be noted that we performed our factor analysis using the Unweighted Least Squares method with oblique Oblimin rotation, since factors were correlated. The Kaiser-Meyer-Olkin (KMO) Measure of Sampling Adequacy was high (KMO = 0.903) and Bartlett’s Test of Sphericity was statistically significant (*p* < 0.001).

**Table 8:** Rotated factor loading matrix of the predefined-factor model.

	Factor							
	1	2	3	4	5	6	7	8
Item	13.2	2.7	1.4	1.3	1.0	0.9	0.7	0.5
21	**0.793**							
22	**0.713**							
20	**0.399**					0.305		
19	**0.315**					0.305		
14		**-0.993**						
13		**-0.828**						
15		**-0.763**						
7			**0.842**					
8			**0.807**					
9			**0.642**					
11				**-0.913**				
12				**-0.790**				
10				**-0.684**				
1					**0.995**			
3					**0.537**			
23						**0.789**		
25						**0.720**		
24						**0.685**		
5							**0.901**	
4							**0.670**	
6							**0.507**	
2					**0.305**		0.305	
17								**0.961**
18								**0.735**
16								**0.734**

**Correlations**								

Factor 1	1.000	**-**0.609	0.511	**-**0.331	0.326	0.604	0.250	0.531
Factor 2	-0.609	1.000	**-**0.485	0.172	**-**0.286	**-**0.397	**-**0.149	**-**0.245
Factor 3	0.511	**-**0.485	1.000	**-**0.491	0.385	0.447	0.471	0.543
Factor 4	**-**0.331	0.172	**-**0.491	1.000	**-**0.437	**-**0.328	**-**0.470	**-**0.565
Factor 5	0.326	**-**0.286	0.385	**-**0.437	1.000	0.353	0.492	0.422
Factor 6	0.604	-0.397	0.447	**-**0.328	0.353	1.000	0.411	0.492
Factor 7	0.250	-0.149	0.471	-0.470	0.492	0.411	1.000	0.402
Factor 8	0.531	**-**0.245	0.543	-0.565	0.422	0.492	0.402	1.000

*Eigenvalues are presented below the names of factors. Lower part of the table shows correlations between the factors. Loadings > 0.30 displayed. Loadings assigned to particular factors in bold.*

**Table 8** presents the pattern matrix of an eight-factor solution. All factors contribute to the loadings of the 25 items and correspond to the respective subscales of the questionnaire. Except for three items, all loadings have values greater than 0.5. The remaining items (TFI 20, TFI 19, and TFI 2) carry loadings higher than 0.3 and are loaded by two factors. The decision to assign item TFI 2 to the intrusive subscale seems arbitrary, since it is equally loaded (0.305) by the factor referring to the sense of control subscale. However, assignment of this item to any other factor would leave only two items remaining in the subscale, which is not recommended (Meikle et al., 2012).

In summary, our investigation of the factor loadings and their corresponding eigenvalues led to the conclusion that the original structure of the questionnaire should be replicated.

For exploratory purposes, we also investigated the results of a factor analysis with orthogonal rotations (Supplementary Tables S12 and S13 show the results). The loadings obtained from the Varimax solution suggest a seven-factorial structure; for the Quartimax solution, the corresponding number reduces to five.

Moreover, we carried out a CFA for investigating if we can confirm the original eight-factorial structure suggested by Meikle et al. (2012). The results, on the one hand, were not fully satisfactory in terms of fit indices and thus did not permit us to conclude an acceptable fit with the data (Supplementary Table S14 presents details on the estimation results). On the other hand, some of these values were not much lower than those obtained by Fackrell et al. (2016); e.g., TLI (0.915 vs. 0.939) or RMSEA (0.084 vs. 0.064). Obviously, the same limitations caused by our comparably low sample size apply.

## Discussion

### Objectives

The main goal of our study was to provide Polish clinicians and researchers with validated translations of the THI and the TFI questionnaires that could facilitate the classification of tinnitus patients according to severity and potential evaluation of therapeutic outcomes. The results of the presented work show that valid and reliable Polish versions of the THI and TFI questionnaires were constructed. The THI-Pl and TFI-Pl are satisfactory in terms of construct and criterion validity. They may be used by Polish clinicians working with people who have tinnitus and by Polish scientists working on international scientific research reports. Moreover, the TFI-Pl provides a decent psychometric tool and may be used by Polish clinicians working with patients suffering from tinnitus by enabling the detection of treatment-related changes. Polish scientists working in the tinnitus field may consider scores and subscores to identify factors potentially influencing results obtained during their research. For the THI-Pl one may focus stronger on the total score in clinical and scientific reports.

### Limitations

We would also like to address two issues, which could be considered as limitations of our study. The size of our sample was not very large (98 patients for the THI-Pl, 108 patients for the TFI-Pl), and that created natural limits, for instance, for performing the CFA which could be of potential interest for some professional researchers working on adaptations or construction of questionnaires. On the other hand, only four among the 19 adaptations of THI cited in this paper were based on larger amounts of questionnaires (Brazilian/Portuguese–180, French–174, German–373, Japanese–182; Shinden et al., 2002; Ferreira et al., 2005; Schmidt et al., 2006; Ghulyan-Bédikian et al., 2010; Kleinstäuber et al., 2015, respectively), and only the German version of the THI included a presentation of the CFA results. In that study the CFA was justified by a sufficient number of patients. Six studies recruited a similar number of participants (Chinese–114, Persian–102, Italian–100, Korean–111, Turkish–110, and Arabic–100; Kim et al., 2002; Aksoy et al., 2007; Monzani et al., 2008; Kam et al., 2009; Jalali et al., 2015; Barake et al., 2016), and the remaining nine studies tested much fewer tinnitus patients. As far as the TFI is concerned, both the Dutch and English adaptations recruited more participants (263 and 294; Fackrell et al., 2014; Rabau et al., 2014, respectively). In the first case, the authors performed an EFA; in the second a CFA. Our aim was to adapt already existing tools in a manner that would enable the comparison of the THI-Pl and the TFI-Pl with other language versions. We believe that our choice of sample size and factor analysis has good justification in light of the mentioned literature. Future work will be needed to assess the test-retest reliability, which, when confirmed would add important value to Polish versions of the THI and TFI questionnaires.

### The Polish version of the THI

Conclusions from the THI-Pl factor analysis are ambiguous since the scree-plot shows a clear dominance of the first factor and limited contribution of the second and third factors in terms of explained variation, which supports a unifactorial structure. On the other hand, multiple factor loadings above the threshold of 0.4 attributed to the second and third factors suggest that a three-factorial structure should not be discarded. Therefore, our results are between those of Newman et al. (1996) and the more recent findings suggesting a unifactorial structure (Baguley and Andersson, 2003; Fackrell et al., 2014). Consequently, one could eventually consider the current subscale division or alternatively simply treat the THI-Pl as a homogenous tool.

Lastly, one aspect is particularly noteworthy in the context of the factor analysis results. Since all factors are moderately correlated with each other, an oblique rotation seems appropriate. However, ignoring the correlation and using the orthogonal Quartimax rotation may lead to substantially different results, with loadings clearly supporting a unifactorial structure. It may be possible that related phenomena may be, in part, responsible for varying results on the factorial structure obtained by different studies.

High correlations between the THI subscales observed in the original version of the questionnaire were also observed in the Polish THI adaptation. Similarly, as far as the total and subscale scores are concerned, the THI-Pl has high internal reliability. Convergent validity was confirmed by strong correlations with the VAS scale accessing tinnitus severity annoyance and the VAS loudness scale. Discriminant validity was assessed by moderate associations between the THI and the CES-D measuring the self-perceived level of depression, and weak associations for the SWLS scale measuring the self-perceived quality of life. Thus, our expectations concerning convergent and discriminant validity were confirmed.

### The Polish version of the TFI

During the development of the first version of the TFI containing 43 items, Meikle et al. (2012) reported four factors with eigenvalues higher or equal to 1 when performing the factor analysis. Nevertheless, they decided to preserve eight subscales in the TFI and continued further analysis on a second prototype and the final version of the questionnaire with a specified number (8) of factors. For statistical purposes only—i.e., considering factors with eigenvalues greater or equal to one—we decided to perform an additional factor analysis with five factors. This approach was also motivated by the scree plot (Supplementary Figure S2), which does not support an eight-factorial structure, but rather suggests three to four factors, thus encouraging the exclusion of factors with eigenvalues smaller than 1. Moreover, the five factors contributed to 78.4% of the explained variation, which can be considered satisfactory. Note that for the sake of comparability with our eight-factor solution, we chose a similar setting (Unweighted Least Squares, oblique Oblimin rotation). In the five-factorial solution the first and main factor loaded 10 items, and the second, third, fourth, and fifth factors loaded five, four, three, and three items, respectively. The relaxation, quality of life, and emotional subscales were assigned to one factor, and the intrusive subscale was separated into sense of control (two items) and sleep (one item) subscales. This observation is in line with concerns described by Meikle et al. (2012). These authors state that the intrusive, quality of life, and emotional subscales may reflect only a general tinnitus severity factor or both general and specific factors. Our conclusions from five- and eight-factorial solutions seem to confirm this reflection. The rotated factor matrix of this solution is available in Supplementary Table S11. Furthermore, for the TFI-Pl both orthogonal transformations investigated suggest factorial structures with a lower number of factors. This suggests, in particular, that the popular Varimax rotation should be used with care for analyzing the TFI-Pl (and potentially the TFI in other languages).

The TFI-Pl presents excellent internal consistency reliability and is satisfactory in terms of convergent and discriminant validity. As for the factor analysis results, an eight-factorial solution assigns all items to appropriate subscales for both the TFI-Pl and the original version of the TFI.

Finally, we would like to emphasize the importance of the fact that Polish versions of tinnitus questionnaires will provide Polish researchers with the possibility to become active within international scientific networks. In light of the complex nature of tinnitus, the collaboration between different communities seems to be of very great importance. Using the THI-Pl and TFI-Pl, Polish scientists will not only be able to compare and report results of their studies dedicated to tinnitus, but also become valued contributors on international scientific platforms.

## Ethics Statement

The study was performed in three ENT centers in Poland, namely Poznan, Gdansk, and Lodz. Heads of these centers (MD Eugeniusz Szymiec, MD Wiesława Klemens and MD Piotr Kotylo) agreed on the procedures and provided access to tinnitus patients. Participation was voluntary and completely anonymous, all procedures were non-intrusive. Every patient gave oral consent before filling out the questionnaire. Patients visiting one of the three ENT centers in Poland were informed about the aim of the study as well as the estimated time for completing the questionnaires. Once a patient agreed to volunteer, a study information sheet was provided.

## Author Contributions

MW translated the questionnaire, designed and performed experiments, analyzed data and wrote the paper; ES, AL-M, and MM performed experiments at the ENT center in Poznań; WK performed experiments at the ENT Private Practice in Gdynia; PK performed experiments at the ENT center in Łódź; WS consulted on the redaction of the manuscript at all stages, and contributed to the revision of the manuscript; AP commented on the manuscript at all stages; JB provided statistical expertise and revised the manuscript at several stages.

## Conflict of Interest Statement

The authors declare that the research was conducted in the absence of any commercial or financial relationships that could be construed as a potential conflict of interest.

## References

[B1] Adamchic I. Langguth B. Hauptmann C. Tass P. A. (2012). Psychometric evaluation of visual analog scale for the assessment of chronic tinnitus. *Am. J. Audiol.* 21 215–225. 10.1044/1059-0889(2012/12-0010) 22846637

[B2] Aksoy S. Firat Y. Alpar R. (2007). The Tinnitus Handicap Inventory: a study of validity and reliability. *Int. Tinnitus J.* 3 94–98. 18229787

[B3] Baguley D. McFerran D. Hall D. (2013). Tinnitus. *Lancet* 382 1600–1607. 10.1016/S0140-6736(13)60142-7 23827090

[B4] Baguley D. M. Andersson G. (2003). Factor analysis of the Tinnitus Handicap Inventory. *Am. J. Audiol.* 12 31–34. 10.1044/1059-0889(2003/007) 12894865

[B5] Barake R. Rizk S. A. Ziade G. Zaytoun G. Bassim M. (2016). Adaptation of the arabic version of the Tinnitus Handicap Inventory. *Otolaryngol. Head Neck Surg.* 154 508–512. 10.1177/0194599815621551 26671903

[B6] Cima R. F. Andersson G. Schmidt C. J. Henry J. A. (2014). Cognitive-behavioral treatments for tinnitus: a review of the literature. *J. Am. Acad. Audiol.* 25 29–61. 10.3766/jaaa.25.1.4 24622860

[B7] Crönlein T. Langguth B. Pregler M. Kreuzer P. M. Wetter T. C. Schecklmann M. (2016). Insomnia in patients with chronic tinnitus: cognitive and emotional distress as moderator variables. *J. Psychosom. Res.* 83 65–68. 10.1016/j.jpsychores.2016.03.001 27020079

[B8] Diener E. Emmons R. A. Larsen R. J. Griffin S. (1985). The satisfaction with life scale. *J. Pers. Assess.* 49 71–75. 10.1207/s15327752jpa4901_13 16367493

[B9] Dojka E. Gorkiewicz M. Pajak A. (2003). Psychometric value of CES-D scale for the assessment of depression in polish population. *Psychiatr. Pol.* 37 281–292. 12776659

[B10] Fackrell K. Hall D. A. Barry J. Hoare D. J. (2014). “Tools for tinnitus measurement: development and validity of questionnaires to assess handicap and treatment effects,” in *Tinnitus: Causes Treatment and Short & Long-Term Health Effects* eds Signorelli F. Turjman F. (New York, NY: Nova Science Publishers Inc) 13–60.

[B11] Fackrell K. Hall D. A. Barry J. G. Hoare D. J. (2016). Psychometric properties of the Tinnitus Functional Index (TFI): assessment in a UK research volunteer population. *Hear. Res.* 335 220–235. 10.1016/j.heares.2015.09.009 26415998 PMC5708524

[B12] Ferreira F. E. Cunha F. Onishi E. T. Barreiro F. Gananca F. (2005). Tinnitus Handicap Inventory: cross-cultural adaptation to Brazilian Portuguese. *Pro Fono* 17 303–310. 10.1590/S0104-56872005000300004 16389787

[B13] Floyd F. J. Widaman K. F. (1995). Factor analysis in the development and refinement of clinical assessment instruments. *Psychol. Assess.* 7 286–299. 10.1037/1040-3590.7.3.286

[B14] Folmer R. L. Theodoroff S. M. Casiana L. Shi Y. Griest S. Vachhani J. (2015). Repetitive transcranial magnetic stimulation treatment for chronic tinnitus: a randomized clinical trial. *JAMA Otolaryngol. Head Neck Surg.* 141 716–722. 10.1001/jamaoto.2015.1219 26181507

[B15] Ghulyan-Bédikian V. Paolino M. Giorgetti-D’Esclercs F. Paolino F. (2010). Propriétés psychométriques d’une version francaise du Tinnitus Handicap Inventory [Psychometric properties of a French adaptation of the Tinnitus Handicap Inventory]. *L’Encéphale.* 36 390–396. 10.1016/j.encep.2009.12.007 21035629

[B16] Jalali M. M. Soleimani R. Fallahi M. Aghajanpour M. Elahi M. (2015). Psychometric properties of the persian version of the Tinnitus Handicap Inventory (THI-P). *Iran J. Otorhinolaryngol.* 27 83–94. 25938079 PMC4409952

[B17] Jastreboff P. J. Hazel J. W. P. (1993). A neurophysiological approach to tinnitus. Clinical implications. *Br. J. Audiol.* 27 7–17. 10.3109/03005369309077884 8339063

[B18] Jastreboff P. J. Hazell J. W. P. (2004). *Tinnitus Retraining Therapy: Implementing the Neurophysiological Model*. Cambridge: Cambridge University Press.

[B19] Juczyński Z. (2001). *Narzędzia Pomiaru w Promocji i Psychologii Zdrowia [Measurement Tools in Health Promotion and Health Psychology]*. Warszawa: Pracownia Testów Psychologicznych Polskiego Towarzystwa Psychologicznego.

[B20] Kam A. C. S. Cheung A. P. P. Chan P. Y. B. Leung E. K. S. Wong T. K. C. Van Hasselt C. A. (2009). Psychometric properties of the Chinese (Cantonese) Tinnitus Handicap Inventory. *Clin. Otolaryngol.* 34 309–315. 10.1111/j.1749-4486.2009.01946.x 19673977

[B21] Kaniasty K. (2003). *Klęska Żywiołowa czy Katastrofa Społeczna? Psychospołeczne Konsekwencje Polskiej Powodzi 1997 Roku [Natural or Social Catastrophy? Psychosocial Consequences of 1997 Flood in Poland]*. Gdańsk: Gdańskie Wydawnictwo Psychologiczne.

[B22] Kehrle H. M. Sampaio A. L. Granjeiro R. C. Oliveira T. S. Oliveira C. A. (2016). Tinnitus annoyance in normal-hearing individuals correlation with depression and anxiety. *Ann. Otol. Rhinol. Laryngol.* 125 185–194. 10.1177/0003489415606445 26424781

[B23] Kim J. H. Lee S. Y. Kim C. H. Lim S. L. Shin J. Chung W. H. (2002). Reliability and validity of a Korean adaptation of the Tinnitus Handicap Inventory. *Korean J. Otolaryngol.* 45 328–334.

[B24] Kleinstäuber M. Frank I. Weise C. (2015). A confirmatory factor analytic validation of the Tinnitus Handicap Inventory. *J. Psychosom. Res.* 78 277–284. 10.1016/j.jpsychores.2014.12.001 25582803

[B25] Krings J. G. Wineland A. Kallogjeri D. Rodebaugh T. L. Nicklaus J. Lenze E. J. (2014). A novel treatment for tinnitus and tinnitus-related cognitive difficulties using computer-based cognitive training and D-Cycloserine. *JAMA Otolaryngol. Head Neck Surg.* 141 18–26. 10.1001/jamaoto.2014.2669 PMC5514293 25356570

[B26] Landgrebe M. Zeman F. Koller M. Eberl Y. (2010). The Tinnitus Research Initiative (TRI) database: a new approach for delineation of tinnitus subtypes and generation of predictors for treatment outcome. *BMC Med. Inform. Decis. Mak.* 10:42 10.1186/1472-6947-10-42 PMC2920857 20682024

[B27] Langguth B. Landgrebe M. Kleinjung T. Sand G. P. Hajak G. (2011). Tinnitus and depression. *World J. Biol. Psychiatry* 12 489–500. 10.3109/15622975.2011.575178 21568629

[B28] Maldonado Fernández M. Shin J. Scherer R. W. Murdin L. (2015). Interventions for tinnitus in adults: an overview of systematic reviews. *Cochrane Database Syst. Rev.* 2015:CD011795 10.1002/14651858.CD011795

[B29] Martines F. Bentivegna D. Martines E. Sciacca V. Martinciglio G. (2010). Characteristics of tinnitus with or without hearing loss: clinical observations in Sicilian tinnitus patients. *Auris Nasus Larynx* 37 685–693. 10.1016/j.anl.2010.03.008 20430549

[B30] Meikle M. B. Henry J. A. Griest S. E. Stewart B. J. Abrams H. B. McArdle R. (2012). The Tinnitus Functional Index: development of a new clinical measure for chronic intrusive tinnitus. *Ear Hear.* 33 153–176. 10.1097/AUD.0b013e31822f67c0 22156949

[B31] Monzani D. Genovese E. Marrara A. Gherpelli C. Pingani L. Forghieri M. (2008). Validity of the Italian adaptation of the Tinnitus Handicap Inventory; focus on quality of life and psychological distress in tinnitus-sufferers. *Acta Otorhinolaryngol. Ital.* 28 126–134. 18646574 PMC2644986

[B32] Newman C. W. Jacobson G. P. Spitzer J. B. (1996). Development of the Tinnitus Handicap Inventory. *Arch Otolayrngol. Head Neck Surg.* 122 143–148. 10.1001/archotol.1996.01890140029007 8630207

[B33] Newman C. W. Sandridge S. A. Jacobson G. P. (1998). Psychometric adequacy of the Tinnitus Handicap Inventory (THI) for evaluating treatment outcome. *J. Am. Acad. Audiol.* 9 153–160. 9564679

[B34] Oron Y. Sergeeva N. Kazlak M. Barbalat I. Spevak S. Lopatin A. S. (2015). Russian adaptation of the Tinnitus Handicap Inventory. *Int. J. Audiol.* 54 485–489. 10.3109/14992027.2014.996823 25620408

[B35] Overdevest J. B. Pross S. E. Cheung S. W. (2015). Tinnitus following treatment for sporadic acoustic neuroma. *Laryngoscope* 126 1639–1643. 10.1002/lary.25672 26403598

[B36] Pavot W. Diener E. (1993). Review of the satisfaction with life scale. *Psychol. Assess.* 5 164–172. 10.1037/1040-3590.5.2.164

[B37] Rabau S. Wouters K. Van de Heyning P. (2014). Validation and translation of the dutch tinnitus functional index. *B-ENT.* 10 251–258. 25654947

[B38] Radloff L. S. (1977). The CES-D Scale: a selfreport depression scale for research in the general population. *Appl. Psychol. Meas.* 1 385–401. 10.1177/014662167700100306

[B39] Raj-Koziak D. Kochanek K. Piłka A. Bartnik G. Fabijańska A. Skarżyński H. (2011). Ocena częstości występowania szumów usznych u dzieci z prawidłowym wynikiem badania przesiewowego słuchu [Tinnitus in children with normal results of hearing screening test]. *Otolaryngologia* 10 171–175.

[B40] Roland L. T. Lenze E. J. Hardin F. M. Kallogjeri D. Nicklaus J. Wineland A. M. (2015). Effects of mindfulness based stress reduction therapy on subjective bother and neural connectivity in chronic tinnitus. *Otolaryngol. Head Neck Surg.* 152 919–926. 10.1177/0194599815571556 25715350 PMC4650869

[B41] Schecklmann M. Vielsmeier V. Steffens T. Landgrebe M. Langguth B. Kleinjung T. (2012). Relationship between audiometric slope and tinnitus pitch in tinnitus patients: insights into the mechanisms of tinnitus generation. *PLoS ONE* 7:e34878 10.1371/journal.pone.0034878 PMC3329543 22529949

[B42] Schmidt L. P. Teixeria V. N. Dall’Igna C. Dallagnol D. Smith M. M. (2006). Brazilian Portuguese language version of the “Tinnitus Handicap Inventory”; validity and reproducibility. *Rev. Bras. Otorrinolaringol.* 72 808–810. 10.1016/S1808-8694(15)31048-X PMC9442134 17308834

[B43] Shekhawat G. S. Searchfield G. D. Kobayashi K. Stinear C. M. (2013). Prescription of hearing-aid output for tinnitus relief. *Int. J. Audiol.* 52 617–625. 10.3109/14992027.2013.799787 23859059

[B44] Shinden S. Ogawa K. Inoue Y. Tazoe M. Asano K. (2002). Tinnitus annoyance and difficulty in activities of daily life evaluated by the Tinnitus Handicap Inventory (THI). *Audiol. Japan.* 45 685–691. 10.4295/audiology.45.685

[B45] Skarżyński H. Rogowski M. Bartnik G. Fabijańska A. (2000). Organization of Tinnitus Management in Poland. *Acta Otolaryngol.* 120 225–226. 10.1080/000164800750000973 11603778

[B46] Tass A. P. Adamchic I. Freund H. J. von Stackelberg T. Hauptmann C. (2012). Counteracting tinnitus by acoustic coordinated reset neuromodulation. *Restor. Neurol. Neurosci.* 30 137–159. 10.3233/RNN-2012-110218 22414611

[B47] Udupi V. A. Uppunda A. K. Mohan K. M. Alex J. Mahendra M. H. (2013). The relationship of perceived severity of tinnitus with depression, anxiety, hearing status, age and gender in individuals with tinnitus. *Int. Tinnitus J.* 18 29–34. 10.5935/0946-5448.20130005 24995897

[B48] Vernon J. A. Meikle M. B. (2003). Tinnitus: clinical measurement. *Otolaryngol. Clin. North Am.* 36 293–305. 10.1016/S0030-6665(02)00162-7 12856298

[B49] Wilson M. B. Kallogjeri D. Joplin C. N. Gorman M. D. Krings J. G. Lenze E. J. (2015). Ecological momentary assessment of tinnitus using smartphone technology. A pilot study. *Otolaryngol. Head Neck Surg.* 152 897–903. 10.1177/0194599815569692 25676150 PMC4580970

[B50] Wise K. Kobayashi K. Searchfield G. D. (2015). Feasibility study of a game integrating assessment and therapy of tinnitus. *J. Neurosci. Methods* 15 1–7. 10.1016/j.jneumeth.2015.04.002 25863140

[B51] Zachariae R. Mirz F. Johansen L. V. Andersen S. E. Bjerring P. Pedersen C. B. (2000). Reliability and validity of a Danish adaptation of the Tinnitus Handicap Inventory. *Scand. Audiol.* 29 37–43. 10.1080/010503900424589 10718675

[B52] Ziarko M. Kaczmarek ŁD. Haładziński P. (2013). Polish version of Center for Epidemiological Studies Depression Scale (CES-D): results of a preliminary study on the psychometric properties of the scale. *Curr. Issues Personal. Psychol.* 1 51–61. 10.5114/cipp.2013.40637

[B53] Zobay O. Palmer A. R. Hall D. A. Sereda M. Adjamian P. (2015). Source space estimation of oscillatory power and brain connectivity in tinnitus. *PLoS ONE* 10:e0120123 10.1371/journal.pone.0120123 PMC4370720 25799178

