# Supplementary material for: Polish Translation and Validation of the Tinnitus Handicap Inventory and the Tinnitus Functional Index
Source: Front Psychol. 2016 Nov 29;7:1871. doi: 10.3389/fpsyg.2016.01871 (PMC5126044; doi:10.3389/fpsyg.2016.01871)
Supplement: Supplementary file 2 [file Table_2.DOCX]

**Table 2**

*Participants that filled in the TFI-Pl – descriptive measures; percentage, missing data excluded.*

| % of responses | Gender | | | | | | | | |  | | | | | | | | | | | | Clinical Center | | | | | | | | | |  |
| --- | --- | --- | --- | --- | --- | --- | --- | --- | --- | --- | --- | --- | --- | --- | --- | --- | --- | --- | --- | --- | --- | --- | --- | --- | --- | --- | --- | --- | --- | --- | --- | --- |
|  | Women | | | | Men | | | | | | |  | | | |  | | | | | Poznań | | | | | Gdańsk | | | Łódź | |  |  |
|  | 54.6 | | | | 45.4 | | | | | | |  | | | |  | | | | | 61.1 | | | | | 12.0 | | | 26.9 | | |  |
|  | Characteristics | | | | | | | | | | |  | |  | | | | | Localization | | | | | | | | | | | | |  |
|  | Tonal | | Noise | | | | Both | | | | |  | | | | | | | | | Left ear | | | | | Right ear | | | Both/centered | | | |
|  | 40.7 | | 51.6 | | | | 7.7 | | | | |  | |  | | | | | 29.0 | | | | | | | 14.0 | | | 57.0 | | |  |
|  | Frequency | | | | | | | | | | | | | | | | | | | | | | | | | | | | | | |  |
|  | 125 | 250 | | | | 500 | | | 1000 | | | | 2000 | | | | | | | 3000 | | | 4000 | | | | 6000 | | | 8000 | |  |
|  | 4.8 | 2.4 | | | | 4.8 | | | 3.6 | | | | 4.8 | | | | | | | 6.0 | | | 18.1 | | | | 10.8 | | | 44.6 | |  |
|  | Hearing | | | | | | |  | | | | | | | Age | | | | | | | | | | | | | | | |  |  |
|  | Normal | | | Loss | | | | | | |  | | | | | | >20 and <=40 | | | | | | | >40 and <=60 | | | | >60 and <=80 | | | |  |
|  | 42.2 | | | 57.8 | | | | | | |  | | | | | | 20.8 | | | | | | | 51.5 | | | | | 27.7 | | |  |
|  | Duration | | | | | | | | | | | | | | | | | | | | | | | | | | | | | | |  |
|  | <2 | | | | >2 and <=4 | | | | | | >4 and <=6 | | | | | | | >6 and <=8 | | | | | | | >8 and <=10 | | | | >10 | | |  |
|  | 36.8 | | | | 18.9 | | | | | | 15.8 | | | | | | | 7.4 | | | | | | | 6.3 | | | | 14.7 | | |  |
