# Supplementary material for: Polish Translation and Validation of the Tinnitus Handicap Inventory and the Tinnitus Functional Index
Source: Front Psychol. 2016 Nov 29;7:1871. doi: 10.3389/fpsyg.2016.01871 (PMC5126044; doi:10.3389/fpsyg.2016.01871)
Supplement: Supplementary file 3 [file Table_3.DOCX]

**Table 3**

*The average endorsement rate (in %) of the particular item for the THI-Pl and the original version of the THI (in brackets).*

| Scores for each item | | | | | | |
| --- | --- | --- | --- | --- | --- | --- |
| Item | 4 | | 2 | | 0 | |
| F 1 | 24.7 | (24) | 49.5 | (49) | 25.8 | (27) |
| F 2 | 24.0 | (35) | 33.3 | (35) | 42.7 | (30) |
| E 3 | 24.7 | (20) | 46.4 | (38) | 28.9 | (42) |
| F 4 | 10.4 | (18) | 44.8 | (25) | 44.8 | (57) |
| C 5 | 16.7 | (17) | 29.2 | (25) | 54.2 | (58) |
| E 6 | 15.3 | (17) | 37.8 | (26) | 46.9 | (57) |
| F 7 | 39.8 | (24) | 28.6 | (38) | 31.6 | (38) |
| C 8 | 52.1 | (60) | 25.0 | (20) | 22.9 | (20) |
| F 9 | 18.6 | (8) | 23.7 | (29) | 57.7 | (63) |
| E 10 | 14.6 | (29) | 38.5 | (37) | 46.9 | (34) |
| C 11 | 11.6 | (14) | 25.3 | (23) | 63.2 | (63) |
| F 12 | 19.8 | (12) | 34.4 | (26) | 45.8 | (62) |
| F 13 | 14.6 | (10) | 30.2 | (32) | 55.2 | (58) |
| E 14 | 18.9 | (22) | 44.2 | (32) | 36.8 | (46) |
| F 15 | 16.3 | (20) | 33.7 | (29) | 50.0 | (51) |
| E 16 | 22.7 | (25) | 40.2 | (38) | 37.1 | (37) |
| E 17 | 12.2 | (26) | 26.5 | (20) | 61.2 | (54) |
| F 18 | 20.8 | (15) | 44.8 | (42) | 34.4 | (43) |
| C 19 | 53.6 | (63) | 28.9 | (18) | 17.5 | (19) |
| F 20 | 25.5 | (18) | 36.7 | (23) | 37.8 | (59) |
| E 21 | 9.4 | (18) | 26.0 | (26) | 64.6 | (56) |
| E 22 | 14.6 | (25) | 30.2 | (26) | 55.2 | (49) |
| C 23 | 18.8 | (11) | 36.5 | (40) | 44.8 | (49) |
| F 24 | 38.1 | (43) | 29.9 | (25) | 32.0 | (32) |
| E 25 | 14.6 | (16) | 31.3 | (20) | 54.2 | (64) |
| Mean | 22.1 | (23.6) | 34.2 | (29.7) | 43.7 | (46.7) |

*Note:* F=functional, E=emotional, C=catastrophic.
