# Supplementary material for: Polish Translation and Validation of the Tinnitus Handicap Inventory and the Tinnitus Functional Index
Source: Front Psychol. 2016 Nov 29;7:1871. doi: 10.3389/fpsyg.2016.01871 (PMC5126044; doi:10.3389/fpsyg.2016.01871)
Supplement: Supplementary file 4 [file Table_4.DOCX]

**Table 4**

*Corrected item total correlation and the Cronbach's alpha if particular item would be deleted for all items of the THI Pl.*

| Item | Corrected Item-Total Correlation | Cronbach's Alpha |
| --- | --- | --- |
|  |  | If Item Deleted |
| F 1 | .706 | .929 |
| F 2 | .303 | .935 |
| E 3 | .514 | .931 |
| F 4 | .514 | .931 |
| C 5 | .673 | .929 |
| E 6 | .668 | .929 |
| F 7 | .405 | .933 |
| C 8 | .481 | .932 |
| F 9 | .720 | .928 |
| E 10 | .682 | .929 |
| C 11 | .486 | .932 |
| F 12 | .720 | .928 |
| F 13 | .627 | .930 |
| E 14 | .711 | .928 |
| F 15 | .536 | .931 |
| E 16 | .694 | .929 |
| E 17 | .628 | .930 |
| F 18 | .495 | .932 |
| C 19 | .246 | .935 |
| F 20 | .674 | .929 |
| E 21 | .713 | .929 |
| E 22 | .743 | .928 |
| C 23 | .632 | .930 |
| F 24 | .266 | .935 |
| E 25 | .748 | .928 |

*Note:* F=functional, E=emotional, C=catastrophic.
