# Supplementary material for: Polish Translation and Validation of the Tinnitus Handicap Inventory and the Tinnitus Functional Index
Source: Front Psychol. 2016 Nov 29;7:1871. doi: 10.3389/fpsyg.2016.01871 (PMC5126044; doi:10.3389/fpsyg.2016.01871)
Supplement: Supplementary file 5 [file Table_5.DOCX]

**Table 5**

*Comparison of internal consistency reliability for THI=PL, original version and other adapted versions^[[1]](#footnote-1)^.*

| Scale |  |  | Country | | | | | | | | | | | | | | | Mean (SD) |
| --- | --- | --- | --- | --- | --- | --- | --- | --- | --- | --- | --- | --- | --- | --- | --- | --- | --- | --- |
|  | PL | EN | PH | H | DK | T | I | C | BP | G | S | K | P | HUN | ARB | RU | F | PH,…, F |
| THI | 0.93 | 0.93 | 0.92 | 0.93 | 0.93 | 0.89 | 0.91 | 0.94 | 0.93 | 0.93 | 0.90 | 0.90 | 0.96 | 0.95 | 0.93 | 0.94 | 0.90 | 0.92 (0.02) |
| THIf | 0.83 | 0.86 | 0.86 | 0.85 | 0.85 | 0.78 | 0.85 | 0.89 | N/A | N/A | 0.85 | 0.91 | 0.91 | 0.90 | 0.86 | N/A | 0.80 | 0.86 (0.04) |
| THIc | 0.70 | 0.68 | 0.63 | 0.74 | 0.74 | 0.80 | Low | 0.64 | N/A | N/A | 0.42 | 0.73 | 0.80 | 0.74 | 0.66 | N/A | 0.49 | 0.67 (0.12) |
| THIe | 0.90 | 0.87 | 0.82 | 0.90 | 0.88 | 0.75 | 0.85 | 0.89 | N/A | N/A | 0.85 | 0.83 | 0.91 | 0.90 | 0.86 | N/A | 0.85 | 0.86 (0.04) |

*Note:* f=functional, c=catastrophic, e=emotional, PL=Polish, EN =English, PH=Filipino, H=Hebrew, DK=Danish, T=Turkish, I=Italian, C=Chinese

(Cantonese), BP=Brazilian Portuguese, G=German, S=Spanish, K=Korean, P=Persian, HUN=Hungarian , ARB=Arabic, RU=Russian, F=French*.*

N/A=not available. Mean and standard deviation calculated in the last column result from all adapted versions, but excluding the THI=PL.

1. The THI has been widely validated and translated, e.g., into Danish (Zachariae et al., 2000), Spanish (Herraiz et al., 2001), Korean (Kim et al., 2002), Japanese (Shinden et al., 2002), Brazilian Portuguese (Ferreira et al., 2005; Schmidt et al., 2006), Turkish (Aksoy et al., 2007), Italian (Monzani et al., 2008), German (Kleinjung et al., 2007), Chinese (Kam et al., 2009), French (Ghulyan-Bédikian et al., 2010), Hebrew (Oron et al., 2011), Filipino (Tobias et al., 2012), Thai (Limviriyakul and [Supavanich](http://www.ncbi.nlm.nih.gov/pubmed?term=Supavanich%20W%5BAuthor%5D&cauthor=true&cauthor_uid=23252210), 2012), Hungarian (Bencsik et al., 2015), Persian (Jalali, 2015), Russian (Oron et al., 2015) and Arabic (Barake et al., 2016).

   References

   Bencsik, B., Tamás, L., Trimmel, K., Stauder, A. (2015). Hungarian adaptation of the Tinnitus Handicap Inventory: reliability and validity. *Eur Arch Otorhinolaryngol.* **272**, 2243–2248. doi: 101007/s00405-014-3138-8.

   Figueiredo, R., Azevedo, A., Oliveira, P. de M. (2009). Correlation analysis of the visual-analogue scale and the Tinnitus Handicap Inventory in tinnitus patients. *Braz J Otorhinolaryngol*. **75**(1), 76-79.

   Herraiz, C., Hernandez, J., Plaza, M., Tapia, C., de los Santos, G. (2001). Evaluación de la incapacidad en pacientes con acúfenos. *Acta Otorrinolaringol Esp*. **52**,534-538.

   Limviriyakul, S., Supavanich, W. (2012). The validity and reliability of Tinnitus Handicap Inventory Thai version. *Journal of the Medical Association of Thailand.* **95**(11), 1433–1440.

   Tobias, C.A., Llanes, E.G.D., Chiong, C. (2012). Validity of a Filipino translation of the Tinnitus Handicap Inventory. *Int Tinnitus J.* **17**(1), 64–69.

   Those references not listed here are provided in the main text. [↑](#footnote-ref-1)
