# Supplementary material for: Polish Translation and Validation of the Tinnitus Handicap Inventory and the Tinnitus Functional Index
Source: Front Psychol. 2016 Nov 29;7:1871. doi: 10.3389/fpsyg.2016.01871 (PMC5126044; doi:10.3389/fpsyg.2016.01871)
Supplement: Supplementary file 6 [file Table_6.DOCX]

**Table 6**

*Orthogonal rotated factor loading matrices of the predefined-factor models. Eigenvalues are presented below the names of factors. The left part of the table presents results obtained by Varimax rotation. The right part of the table shows results obtained by Quartimax rotation for comparison.*

| Varimax rotation | | | | Quartimax rotation | | | |
| --- | --- | --- | --- | --- | --- | --- | --- |
| Factor | | | | Factor | | | |
| Scale/  Item | 1 | 2 | 3 | Scale/  Item | 1 | 2 | 3 |
|  | 10.25 | 1.88 | 1.38 |  | 10.25 | 1.88 | 1.38 |
| E 16 | **.669** |  | .307 | E 25 | **.777** |  |  |
| F 15 | **.645** |  |  | E 22 | **.777** |  |  |
| F 20 | **.594** | .426 |  | E 21 | **.743** |  | .444 |
| F 7 | **.553** |  |  | E 16 | **.738** |  |  |
| F 18 | **.537** |  | .309 | E 14 | **.738** |  |  |
| F 1 | **.515** | .343 | .366 | F 12 | **.725** |  |  |
| E 10 | **.513** |  | .412 | F 20 | **.717** |  |  |
| E 25 | **.505** | .480 | .348 | F 9 | **.716** | .357 |  |
| C 8 | **.505** |  |  | F 1 | **.715** |  |  |
| E 14 | **.491** | .397 | .386 | E 10 | **.702** |  |  |
| E 6 | **.474** | .327 | .375 | C 5 | **.696** |  |  |
| C 19 | **.393** |  |  | E 6 | **.682** |  |  |
| E 21 |  | **.786** |  | C 23 | **.664** |  |  |
| E 22 | .450 | **.623** |  | F 13 | **.620** | .482 |  |
| F 24 |  | **.526** |  | F 15 | **.583** |  | -.393 |
| C 5 | .423 | **.506** |  | E 3 | **.534** |  |  |
| C 23 | .473 | **.493** |  | F 18 | **.526** |  | -.324 |
| F 4 |  | **.425** | .353 | C 11 | **.512** |  |  |
| C 11 |  | **.410** |  | F 4 | **.511** |  |  |
| E 3 |  | **.360** |  | C 8 | **.506** |  |  |
| E 17 |  |  | **.833** | F 7 | **.474** | -.303 |  |
| F 13 |  |  | **.715** | C 19 | **.281** |  |  |
| F 9 |  | .450 | **.628** | E 17 | .612 | **.631** |  |
| F 12 | .354 | .436 | **.522** | F 2 |  | **.360** |  |
| F 2 |  |  | **.440** | F 24 |  |  | **.497** |

*Note:* Loadings >0.30 displayed (with exception of Item 19 in Quartimax solution). Loadings assigned to particular factors in bold; F=functional, E=emotional, C=catastrophic.
