# Supplementary material for: Polish Translation and Validation of the Tinnitus Handicap Inventory and the Tinnitus Functional Index
Source: Front Psychol. 2016 Nov 29;7:1871. doi: 10.3389/fpsyg.2016.01871 (PMC5126044; doi:10.3389/fpsyg.2016.01871)
Supplement: Supplementary file 7 [file Table_7.DOCX]

**Table 7**

*Mean rates and standard deviations of particular items for the TFI-Pl (on the left) and the original version of the TFI (on the right).*

| Item | TFI-Pl | TFI |
| --- | --- | --- |
| 1 | 5.58±3.23 | 7.2 ± 2.9 |
| 2 | 5.27±2.31 | 6.8 ± 2.3 |
| 3 | 4.97±3.14 | 6.3 ± 3.1 |
| 4 | 6.74±3.22 | 6.9 ± 3.2 |
| 5 | 6.44±3.07 | 5.6 ± 2.7 |
| 6 | 6.06±3.07 | 7.0 ± 2.8 |
| 7 | 4.39±2.76 | 5.1 ± 3.0 |
| 8 | 3.67±2.64 | 4.6 ± 3.1 |
| 9 | 3.98±2.70 | 4.6 ± 3.0 |
| 10 | 4.63±3.39 | 5.4 ± 3.6 |
| 11 | 4.06±3.46 | 5.0 ± 3.7 |
| 12 | 4.08±3.48 | 5.0 ± 3.7 |
| 13 | 4.30±3.05 | 5.3 ± 3.1 |
| 14 | 3.82±3.12 | 5.2 ± 3.1 |
| 15 | 3.88±3.20 | 5.4 ± 3.2 |
| 16 | 4.51±3.09 | 5.9 ± 3.1 |
| 17 | 4.90±3.00 | 5.6 ± 3.2 |
| 18 | 5.72±3.16 | 6.4 ± 3.2 |
| 19 | 3.56±3.19 | 4.8 ± 3.4 |
| 20 | 4.21±3.30 | 5.0 ± 3.2 |
| 21 | 3.54±3.20 | 4.5 ± 3.2 |
| 22 | 3.20±3.09 | 4.1 ± 3.3 |
| 23 | 4.70±3.08 | 4.9 ± 3.3 |
| 24 | 4.64±2.93 | 5.4 ± 3.4 |
| 25 | 4.33±3.09 | 3.8 ± 3.4 |
