# Supplementary material for: Polish Translation and Validation of the Tinnitus Handicap Inventory and the Tinnitus Functional Index
Source: Front Psychol. 2016 Nov 29;7:1871. doi: 10.3389/fpsyg.2016.01871 (PMC5126044; doi:10.3389/fpsyg.2016.01871)
Supplement: Supplementary file 8 [file Table_8.DOCX]

**Table 8**

*Correlations (Spearman’s rho, p) of all scales, age and duration.*

| Variable | THI  -Pl | THI  f | THI  c | THI  e | TFI  -Pl | TFI  int | TFI  soc | TFI  cog | TFI  sleep | TFI  aud | TFI  relax | TFI  qol | TFI  em | VAS  l | VAS  a | SWLS | CES-D | Age | Dur |
| --- | --- | --- | --- | --- | --- | --- | --- | --- | --- | --- | --- | --- | --- | --- | --- | --- | --- | --- | --- |
| THI-Pl | 1 | .933 | .813 | .928 | .782 | .616 | .558 | .639 | .609 | .468 | .606 | .709 | .774 | .597 | .795 | -.301 | .509 | * | .110 |
|  |  | *** | *** | *** | *** | *** | *** | *** | *** | *** | *** | *** | *** | *** | *** | ** | *** | .758 | .314 |
| THIf | .933 | 1 | .642 | .798 | .777 | .575 | .457 | .656 | .636 | .575 | .595 | .736 | .707 | .558 | .719 | -.261 | .508 | .058 | .175 |
|  | *** |  | *** | *** | *** | *** | *** | *** | *** | *** | *** | *** | *** | *** | *** | * | *** | .585 | .107 |
| THIc | .813 | .642 | 1 | .681 | .642 | .580 | .638 | .464 | .451 | .266 | .499 | .508 | .712 | .553 | .719 | -.248 | .333 | ** | * |
|  | *** | *** |  | *** | *** | *** | *** | *** | *** | * | *** | *** | *** | *** | *** | * | ** | .979 | .763 |
| THIe | .928 | .798 | .681 | 1 | .657 | .498 | .478 | .550 | .497 | .354 | .507 | .618 | .697 | .498 | .682 | -.347 | .496 | .019 | .074 |
|  | *** | *** | *** |  | *** | *** | *** | *** | *** | ** | *** | *** | *** | *** | *** | ** | *** | .860 | .495 |
| TFI-Pl | .782 | .777 | .642 | .657 | 1 | .761 | .675 | .803 | .734 | .608 | .793 | .864 | .816 | .761 | .837 | -.223 | .361 | .105 | .124 |
|  | *** | *** | *** | *** |  | *** | *** | *** | *** | *** | *** | *** | *** | *** | *** | * | *** | .304 | .240 |
| TFIint | .616 | .575 | .580 | .498 | .761 | 1 | .638 | .561 | .581 | .352 | .568 | .559 | .577 | .724 | .666 | -.156 | .180 | .123 | .162 |
|  | *** | *** | *** | *** | *** |  | *** | *** | *** | *** | *** | *** | *** | *** | *** | .130 | .074 | .229 | .126 |
| TFIsoc | .558 | .457 | .638 | .478 | .675 | .638 | 1 | .539 | .539 | .224 | .529 | .484 | .577 | .584 | .656 | -.257 | .183 | .101 | ** |
|  | *** | *** | *** | *** | *** | *** |  | *** | *** | * | *** | *** | *** | *** | *** | * | .070 | .324 | .982 |
| TFIcog | .639 | .656 | .464 | .550 | .803 | .561 | .539 | 1 | .602 | .584 | .632 | .722 | .653 | .617 | .691 | -.309 | .469 | .055 | .180 |
|  | *** | *** | *** | *** | *** | *** | *** |  | *** | *** | *** | *** | *** | *** | *** | ** | *** | .594 | .088 |
| TFIsleep | .609 | .636 | .451 | .497 | .734 | .581 | .539 | .602 | 1 | .276 | .616 | .521 | .537 | .583 | .633 | -.264 | .394 | .111 | .068 |
|  | *** | *** | *** | *** | *** | *** | *** | *** |  | ** | *** | *** | *** | *** | *** | * | *** | .277 | .521 |
| TFIaud | .468 | .575 | .266 | .354 | .608 | .352 | .224 | .584 | .276 | 1 | .305 | .689 | .504 | .282 | .365 | -.016 | .259 | .218 | .342 |
|  | *** | *** | * | ** | *** | *** | * | *** | ** |  | ** | *** | *** | * | ** | .878 | * | * | ** |
| TFIrelax | .606 | .595 | .499 | .507 | .793 | .568 | .529 | .632 | .616 | .305 | 1 | .643 | .621 | .633 | .658 | -.167 | .261 | * | * |
|  | *** | *** | *** | *** | *** | *** | *** | *** | *** | ** |  | *** | *** | *** | *** | .105 | ** | .777 | .741 |
| TFIqol | .709 | .736 | .508 | .618 | .864 | .559 | .484 | .722 | .521 | .689 | .643 | 1 | .822 | .617 | .695 | -.193 | .389 | .127 | .173 |
|  | *** | *** | *** | *** | *** | *** | *** | *** | *** | *** | *** |  | *** | *** | *** | .061 | *** | .213 | .100 |
| TFIem | .774 | .707 | .712 | .697 | .816 | .577 | .577 | .653 | .537 | .504 | .621 | .822 | 1 | .570 | .728 | -.312 | .424 | .057 | .115 |
|  | *** | *** | *** | *** | *** | *** | *** | *** | *** | *** | *** | *** |  | *** | *** | ** | *** | .582 | .280 |

*Note:* THI=Tinnitus Handicap Inventory, Pl=Polish, f=functional, c=catastrophic, e=emotional, TFI=Tinnitus Functional Index, int=intrusive, soc=sence of control, cog=cognitive, aud=auditory, relax=relaxation, qol=quality of life, em=emotional, VAS=Visual Analog Scale, a=annoyance, l=loudness, CES-D=The Centre for Epidemiological Studies Depression Scale, SWLS=The Satisfaction With Life Scale
