# Supplementary material for: Polish Translation and Validation of the Tinnitus Handicap Inventory and the Tinnitus Functional Index
Source: Front Psychol. 2016 Nov 29;7:1871. doi: 10.3389/fpsyg.2016.01871 (PMC5126044; doi:10.3389/fpsyg.2016.01871)
Supplement: Supplementary file 9 [file Table_9.DOCX]

**Table 9**

*Corrected item total correlation and the Cronbach's alpha if particular item would be deleted for all items of the TFI-Pl.*

| Item | Corrected Item-Total Correlation | Cronbach's Alpha |
| --- | --- | --- |
|  |  | if Item Deleted |
| 1 | .585 | .959 |
| 2 | .736 | .958 |
| 3 | .666 | .959 |
| 4 | .374 | .961 |
| 5 | .669 | .959 |
| 6 | .717 | .958 |
| 7 | .747 | .958 |
| 8 | .740 | .958 |
| 9 | .772 | .958 |
| 10 | .609 | .959 |
| 11 | .655 | .959 |
| 12 | .642 | .959 |
| 13 | .604 | .959 |
| 14 | .487 | .960 |
| 15 | .599 | .959 |
| 16 | .739 | .958 |
| 17 | .715 | .958 |
| 18 | .728 | .958 |
| 19 | .769 | .958 |
| 20 | .833 | .957 |
| 21 | .780 | .957 |
| 22 | .721 | .958 |
| 23 | .784 | .957 |
| 24 | .798 | .957 |
| 25 | .798 | .957 |
