# Supplementary material for: Polish Translation and Validation of the Tinnitus Handicap Inventory and the Tinnitus Functional Index
Source: Front Psychol. 2016 Nov 29;7:1871. doi: 10.3389/fpsyg.2016.01871 (PMC5126044; doi:10.3389/fpsyg.2016.01871)
Supplement: Supplementary file 10 [file Table_10.DOCX]

**Table 10**

*Internal consistency reliability for TFI-Pl and its subscales and different adapted versions.*

| Scale | TFI-Pl | Original | TFI-NZ | TFI-D |
| --- | --- | --- | --- | --- |
| TFI total | 0.96 | 0.97 | 0.97 | 0.95 |
| TFI intrusive | 0.84 | 0.85 | 0.82 | 0.81 |
| TFI sense of control | 0.83 | 0.82 | 0.80 | 0.81 |
| TFI cognitive | 0.92 | 0.96 | 0.97 | 0.94 |
| TFI sleep | 0.90 | 0.97 | 0.95 | 0.92 |
| TFI auditory | 0.93 | 0.97 | 0.97 | 0.91 |
| TFI relaxation | 0.93 | 0.96 | 0.94 | 0.89 |
| TFI quality of life | 0.93 | 0.93 | 0.93 | 0.95 |
| TFI emotional | 0.95 | 0.94 | 0.93 | 0.92 |

*Note:* Pl=Polish, NZ=New Zealand, D=Dutch.
