# Supplementary material for: Polish Translation and Validation of the Tinnitus Handicap Inventory and the Tinnitus Functional Index
Source: Front Psychol. 2016 Nov 29;7:1871. doi: 10.3389/fpsyg.2016.01871 (PMC5126044; doi:10.3389/fpsyg.2016.01871)
Supplement: Supplementary file 11 [file Table_11.DOCX]

**Table 11**

*Rotated factor loading matrix of the five-factor model, Oblimin rotation. Eigenvalues are presented below the names of factors.*

|  | | | | | |
| --- | --- | --- | --- | --- | --- |
|  | Factor | | | | |
|  | 1 | 2 | 3 | 4 | 5 |
| Item | 13.2 | 2.7 | 1.4 | 1.3 | 1.0 |
| 23 | **.925** |  |  |  |  |
| 25 | **.872** |  |  |  |  |
| 24 | **.850** |  |  |  |  |
| 20 | **.815** |  |  |  |  |
| 19 | **.687** |  |  |  |  |
| 22 | **.662** |  |  |  |  |
| 21 | **.660** |  |  |  |  |
| 17 | **.480** | .324 | .351 |  | .317 |
| 18 | **.460** | .311 | .335 |  |  |
| 16 | **.457** |  | **.**313 |  |  |
| 14 |  | **-.826** |  |  |  |
| 13 |  | **-.704** |  |  |  |
| 15 |  | **-.698** |  |  |  |
| 8 |  |  | **.798** |  |  |
| 7 |  |  | **.773** |  |  |
| 9 |  |  | **.743** |  |  |
| 5 |  |  |  | **.650** |  |
| 4 |  |  |  | **.597** |  |
| 6 |  |  | .341 | **.447** |  |
| 1 |  |  |  | **.402** | .365 |
| 2 |  |  | .360 | **.372** |  |
| 11 |  |  |  |  | **.945** |
| 12 |  |  |  |  | **.924** |
| 10 |  |  |  |  | **.580** |
| 3 |  |  |  | .353 | **.439** |

*Note:* Loadings >0.30 displayed. Loadings assigned to particular factors in bold; F=functional, E=emotional, C=catastrophic.
