# Supplementary material for: Polish Translation and Validation of the Tinnitus Handicap Inventory and the Tinnitus Functional Index
Source: Front Psychol. 2016 Nov 29;7:1871. doi: 10.3389/fpsyg.2016.01871 (PMC5126044; doi:10.3389/fpsyg.2016.01871)
Supplement: Supplementary file 12 [file Table_12.DOCX]

**Table 12**

*Rotated factor loading matrix for the Varimax rotation. Eigenvalues are presented below the names of factors.*

|  | Factor | | | | | | | |
| --- | --- | --- | --- | --- | --- | --- | --- | --- |
|  | 1 | 2 | 3 | 4 | 5 | 6 | 7 | 8 |
| Item | 13.2 | 2.7 | 1.4 | 1.3 | 1.0 | 0.9 | 0.7 | 0.5 |
| 23 | **.821** |  |  |  |  |  |  |  |
| 25 | **.807** |  |  |  |  |  |  |  |
| 24 | **.762** |  |  |  |  |  |  |  |
| 20 | **.610** | .308 | .416 |  |  |  |  |  |
| 19 | **.563** | .459 |  |  |  |  |  |  |
| 21 | **.555** | .464 |  |  |  |  |  | .506 |
| 22 | **.500** | .396 |  |  |  |  |  | .464 |
| 14 |  | **.940** |  |  |  |  |  |  |
| 15 |  | **.822** |  |  |  |  |  |  |
| 13 |  | **.802** |  |  |  |  |  |  |
| 17 |  |  | **.839** |  |  |  |  |  |
| 18 |  |  | **.697** |  |  |  |  |  |
| 16 |  |  | **.683** |  |  |  |  |  |
| 11 |  |  |  | **.849** |  |  |  |  |
| 12 |  |  | .326 | **.764** |  |  |  |  |
| 10 |  |  |  | **.671** |  |  |  |  |
| 5 |  |  |  |  | **.847** |  |  |  |
| 4 |  |  |  |  | **.632** |  |  |  |
| 6 |  |  |  |  | **.572** | .309 |  |  |
| 2 |  |  |  |  | **.418** | .368 | .367 |  |
| 7 |  |  |  |  |  | **.734** |  |  |
| 8 |  | .339 |  |  |  | **.715** |  |  |
| 9 |  | .310 |  |  |  | **.619** |  |  |
| 1 |  |  |  |  |  |  | **.894** |  |
| 3 |  |  |  | .320 | .311 |  | **.546** |  |

*Note:* Loadings >0.30 displayed. Loadings assigned to particular factors in bold.
