# Supplementary material for: Polish Translation and Validation of the Tinnitus Handicap Inventory and the Tinnitus Functional Index
Source: Front Psychol. 2016 Nov 29;7:1871. doi: 10.3389/fpsyg.2016.01871 (PMC5126044; doi:10.3389/fpsyg.2016.01871)
Supplement: Supplementary file 13 [file Table_13.DOCX]

**Table 13**

*Rotated factor loading matrix for the Quartimax rotation. Eigenvalues are presented below the names of factors.*

|  | Factor | | | | | | | |
| --- | --- | --- | --- | --- | --- | --- | --- | --- |
|  | 1 | 2 | 3 | 4 | 5 | 6 | 7 | 8 |
| Item | 13.2 | 2.7 | 1.4 | 1.3 | 1.0 | 0.9 | 0.7 | 0.5 |
| 20 | **.894** |  |  |  |  |  |  |  |
| 25 | **.865** |  |  |  |  |  |  |  |
| 24 | **.862** |  |  |  |  |  |  |  |
| 23 | **.857** |  |  |  |  |  |  | -.347 |
| 21 | **.845** |  |  |  |  |  |  |  |
| 19 | **.809** |  |  |  |  |  |  |  |
| 22 | **.790** |  |  |  |  |  |  |  |
| 17 | **.769** |  |  |  |  |  | .549 |  |
| 18 | **.765** |  |  |  |  |  | .399 |  |
| 16 | **.764** |  |  |  |  |  | .399 |  |
| 9 | **.761** |  |  |  |  | .399 |  |  |
| 8 | **.742** |  |  |  |  | .499 |  |  |
| 7 | **.740** |  |  |  |  | .536 |  |  |
| 6 | **.684** |  |  |  |  |  |  |  |
| 2 | **.678** |  |  |  |  |  |  |  |
| 3 | **.616** |  |  |  | .450 |  |  |  |
| 10 | **.572** |  | .542 |  |  |  |  |  |
| 14 | .451 | **.844** |  |  |  |  |  |  |
| 13 | .547 | **.690** |  |  |  |  |  |  |
| 15 | .581 | **.681** |  |  |  |  |  |  |
| 11 | .593 |  | **.725** |  |  |  |  |  |
| 12 | .589 |  | **.647** |  |  |  |  |  |
| 5 | .628 |  |  | **.712** |  |  |  |  |
| 4 | .340 |  |  | **.570** |  |  |  |  |
| 1 | .540 |  |  |  | .**815** |  |  |  |

*Note:* Loadings >0.30 displayed. Loadings assigned to particular factors in bold.
