# Supplementary material for: Polish Translation and Validation of the Tinnitus Handicap Inventory and the Tinnitus Functional Index
Source: Front Psychol. 2016 Nov 29;7:1871. doi: 10.3389/fpsyg.2016.01871 (PMC5126044; doi:10.3389/fpsyg.2016.01871)
Supplement: Supplementary file 14 [file Table_14.DOCX]

**Table 14**

*Model fit statistics of the THI-Pl and the TFI-Pl .*

|  | THI-Pl | TFI-Pl |
| --- | --- | --- |
| χ2 | 521.843 | 424,548 |
| df | 272 | 247 |
| p | <0.001 | <0.001 |
| CMIN/DF | 1.919 | 1.719 |
| RMR | 0.179 | 0.548 |
| GFI | 0.701 | 0.774 |
| TLI | 0.772 | 0.915 |
| RMSEA | 0.097 | 0.084 |

Note: χ2=Chi-square, df=degrees of freedom, p=probability level; Fit measures: CMIN/DF=χ2/df ratio, RMR=root mean square residual, GFI=goodness-of-fit index, TLI=Tucker-Lewis index, RMSEA=root mean square error approximation.
