# Supplementary material for: Polish Translation and Validation of the Tinnitus Handicap Inventory and the Tinnitus Functional Index
Source: Front Psychol. 2016 Nov 29;7:1871. doi: 10.3389/fpsyg.2016.01871 (PMC5126044; doi:10.3389/fpsyg.2016.01871)
Supplement: Supplementary file 15 [file Data_Sheet_1.DOC]

**Polish translation of the Tinnitus Handicap Inventory and the Tinnitus Functional Index**

The following page contains the Polish translation of the Tinnitus Handicap Inventory (THI-Pl). When using the THI, please cite the principle article as source, and do not modify the contents of the form. The usage of the THI-Pl is free of charge, and does not require further approval. We thank Dr. Graig Newman for his permission to develop the Polish translation of the THI.

The Tinnitus Functional Index (TFI) was translated under a non-exclusive, non-sublicensable, and non-transferable license agreement with Oregon Health & Science University (OHSU), Portland OR, USA. The Polish version of the Tinnitus Functional Index (TFI-Pl), is available upon request from OHSU at the following site: <http://www.ohsu.edu/tech-transfer/portal/technology.php?technology_id=1004796>

Imię/ID: ________________________________________________ Data:_____________________

Instrukcje: **Celem kwestionariusza jest identyfikacja trudności, których może Pan/Pani doświadczać**

**z powodu swoich szumów usznych. Proszę odpowiedzieć *Tak*, *Czasami* lub *Nie* na każde pytanie. Proszę nie pominąć żadnego pytania.**

F-1 Czy z powodu Pana/i szumów usznych jest Panu/i trudno się koncentrować? Tak Czasami Nie

F-2 Czy głośność Pana/i szumów usznych utrudnia Panu/i słyszenie ludzi? Tak Czasami Nie

E-3 Czy Pana/i szumy uszne wywołują w Panu/i złość? Tak Czasami Nie

F-4 Czy Pana/i szumy uszne sprawiają, że czuje się Pan/i zdezorientowany/a? Tak Czasami Nie

C-5 Czy Pana/i szumy uszne sprawiają, że czuje się Pana/i zdesperowany/a? Tak Czasami Nie

E-6 Czy z powodu Pana/i szumów usznych bardzo dużo Pan/i narzeka? Tak Czasami Nie

F-7 Czy ma Pan/i problemy z zasypianiem w nocy z powodu Pana/i szumów usznych?

Tak Czasami Nie

C-8 Czy czuje się Pan/i tak, jakby nie mógł/mogła uciec przed swoimi szumami usznymi?

Tak Czasami Nie

F-9 Czy Pana/i szumy uszne przeszkadzają w Pana/i zdolności do cieszenia się ze swoich towarzyskich

aktywności? (takich jak wyjście na kolację, do kina, itd.)? Tak Czasami Nie

E-10 Czy z powodu Pana/i szumów usznych czuje się Pan/i sfrustrowany/a? Tak Czasami Nie

C-11 Czy z powodu Pana/i szumów usznych uważa Pan/i, że ma Pan/i straszną chorobę?

Tak Czasami Nie

F-12 Czy Pana/i szumy uszne utrudniają Panu/i cieszenie się życiem? Tak Czasami Nie

F-13 Czy Pana/i szumy uszne przeszkadzają w Pana/i pracy lub w obowiązkach domowych?

Tak Czasami Nie

E-14 Czy wydaje się Panu/i, że jest z powodu Pana/i szumów usznych często podirytowany/a?

Tak Czasami Nie

F-15 Czy jest Panu/i trudno czytać z powodu Pana/i szumów usznych? Tak Czasami Nie

E-16 Czy Pana/i szumy uszne sprawiają, że Pan/i źle się czuje? Tak Czasami Nie

E-17 Czy czuje Pana/i, że Pana/i problem z szumami usznymi utrudnił Pana/i relacje z członkami Pana/i rodziny i przyjaciółmi? Tak Czasami Nie

F-18 Czy wydaje się Panu/i trudnym odciągnąć swoją uwagę od swoich szumów usznych i przenieść ją na inne rzeczy? Tak Czasami Nie

C-19 Czy czuje Pan/i, że nie ma kontroli nad swoimi szumami usznymi? Tak Czasami Nie

F-20 Czy z powodu swoich szumów usznych często czuje się Pan/i zmęczony/a? Tak Czasami Nie

E-21 Czy z powodu swoich szumów usznych często ma Pan/i poczucie depresji? Tak Czasami Nie

E-22 Czy Pana/i szumy uszne sprawiają, że ma Pan/i poczucie silnego niepokoju? Tak Czasami Nie

C-23 Czy czuje Pan/i, że już dłużej nie może radzić sobie ze swoimi szumami usznymi?

Tak Czasami Nie

F-24 Czy Pana/i szumy uszne pogarszają się, kiedy jest Pan/i pod wpływem stresu? Tak Czasami Nie

E-25 Czy Pana/i szumy uszne sprawiają, że nie ma Pan/i poczucia bezpieczeństwa? Tak Czasami Nie

*F __________ C __________ E __________ T __________*
