# Supplementary figures and images for: Polish Translation and Validation of the Tinnitus Handicap Inventory and the Tinnitus Functional Index
Source: Front Psychol. 2016 Nov 29;7:1871. doi: 10.3389/fpsyg.2016.01871 (PMC5126044; doi:10.3389/fpsyg.2016.01871)

**Figure 1** Scree plots presenting factors and corresponding eigenvalues for THI-Pl.

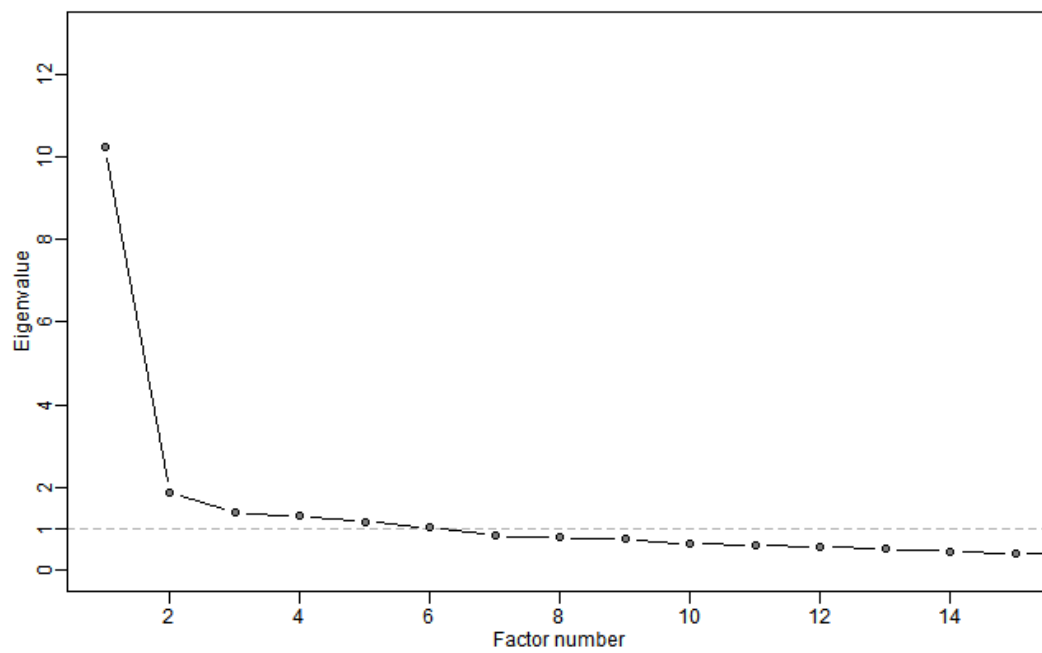

Supplement: Supplementary file 16 [file Image_1.PDF]

**Figure 2** Scree plots presenting factors and corresponding eigenvalues for TFI-PI.

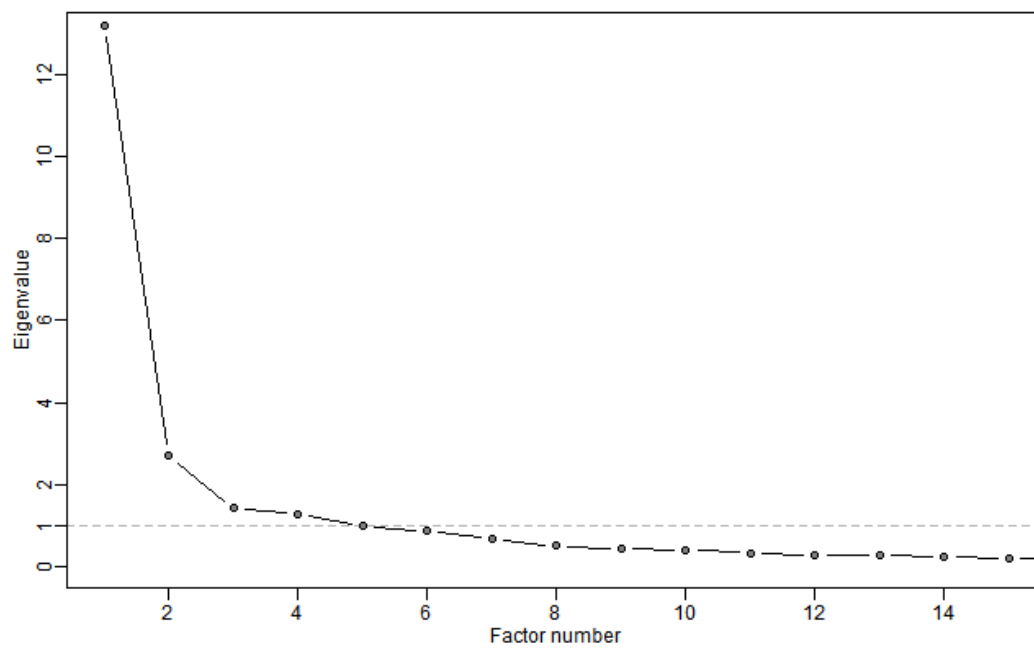

Supplement: Supplementary file 17 [file Image_2.PDF]
